# Outcomes and predictors of second haploidentical hematopoietic stem cell transplantation for pediatric acute leukemia relapsing after a first allograft

**DOI:** 10.3389/fimmu.2026.1885224

**Published:** 2026-07-20

**Authors:** Kai Chen, Huifang Wang, Qing Liu, Guanhua Hu, Zhang Feng, Bing Zou, Yingxi Zuo, Lu Bai, Pan Suo, Jingbo Shao, Hui Jiang, Xiaohui Zhang, Xiaojun Huang, Jinjin Chen, Yifei Cheng

**Affiliations:** 1 Department of Hematology and Oncology, Shanghai Children’s Hospital, School of Medicine, Shanghai Jiao Tong University, Shanghai, China; 2 Peking University People’s Hospital & Peking University Institute of Hematology, National Clinical Research Center for Hematologic Disease, Beijing Key Laboratory of Hematopoietic Stem Cell Transplantation, Beijing, China; 3 Department of Child Health Care, Shanghai Children’s Hospital, School of Medicine, Shanghai Jiao Tong University, Shanghai, China

**Keywords:** graft-versus-leukemia effect, haploidentical hematopoietic stem cell transplantation (haplo-HSCT), pediatric relapsed acute leukemia, prognostic factors, second allogeneic transplantation, survival outcomes

## Abstract

**Background:**

The clinical efficacy and prognostic factors of haploidentical hematopoietic stem cell transplantation (haplo-HSCT) as a salvage approach for children with relapsed acute leukemia (AL) after a first allogeneic HSCT (allo-HSCT) remain unclear. This study aimed to evaluate the clinical efficacy, survival outcomes, and prognostic factors of second haploidentical HSCT in children with relapsed AL following prior allo-HSCT.

**Methods:**

This retrospective study included 40 pediatric patients (median age: 8.5 years) who underwent a second haplo-HSCT at two Chinese transplantation centers between January 2017 and December 2023. The primary endpoints were overall survival (OS) and leukemia-free survival (LFS); and secondary endpoints included cumulative relapse incidence and non-relapse mortality (NRM).

**Results:**

With a median follow-up of 16.0 months, 1- and 2-year OS rates were 77.8% and 65.5%, and LFS rates were 72.4% and 61.0%, respectively. The 2-year cumulative incidences of relapse and NRM were 26.6% and 15.1%, respectively. Multivariate analysis showed that B-cell acute lymphoblastic leukemia was associated with the most favorable 2-year OS (HR = 0.081, 95%CI: 0.007–0.877, P = 0.039). Grade III–IV acute GVHD occurred in 25.0% of patients, and chronic GVHD occurred in 50.0% (10.0% extensive).

**Conclusions:**

These findings suggest that second haplo-HSCT with myeloablative conditioning may be a viable and effective salvage option for pediatric patients with relapsed AL lacking matched donors, possibly through graft-versus-leukemia effects. Future studies should incorporate targeted therapies and immune modulation strategies to improve outcomes in this high-risk population.

## 1 Introduction

Acute leukemia (AL) is one of the most common and aggressive hematologic malignancies in children. Allogeneic hematopoietic stem cell transplantation (allo-HSCT) is a potentially curative treatment for high-risk or relapsed pediatric AL. However, despite advances in transplant protocols and supportive care, relapse after the first allo-HSCT remains a major cause of treatment failure, occurring in approximately 30%–35% of patients (1–3). Post-transplantation relapse is associated with dismal outcomes and presents a formidable clinical challenge, given the limited efficacy of conventional therapies and the high risk of treatment-related morbidity and mortality.

A second allo-HSCT is often considered a potentially salvage option for relapsed patients, particularly those who achieve a second remission. Nevertheless, the success of this strategy is influenced by multiple factors, including the availability of a suitable donor, the intensity of the conditioning regimen, and the risk of graft-versus-host disease (GVHD). In recent years, haploidentical HSCT (haplo-HSCT) has gained increasing attention as a feasible alternative, especially in the setting of urgent need and donor scarcity. Its advantages include immediate donor availability and the potential to harness the graft-versus-leukemia (GVL) effect (4–7).

Despite its growing use, the long-term outcomes of second haplo-HSC for relapsed pediatric AL remain poorly defined. In particular, prognostic factors that could guide risk stratification and treatment optimization are not well established in this setting. Thus, to address this gap, we conducted a multicenter retrospective study to evaluate the clinical efficacy of second haplo-HSCT in children with relapsed AL following initial allo-HSCT, and to identify key prognostic indicators that may inform personalized therapeutic strategies.

## 2 Methods

### 2.1 Study design and participants

This retrospective multicenter study included 40 pediatric patients diagnosed with AL who experienced relapse after a first allo-HSCT and subsequently underwent a salvage haplo-HSCT as a second transplant. Patients were treated at one of two tertiary transplantation centers in China: The Institute of Hematology at Peking University People’s Hospital and the Department of Pediatric Hematology-Oncology at Shanghai Children’s Hospital, Shanghai Jiao Tong University.

Eligible patients were identified retrospectively between January 2017 and December 2023. Patients were included if they met all of the following criteria: 1) age < 18 years at the time of transplantation; 2) diagnosis of acute leukemia, including acute myeloid leukemia or acute lymphoblastic leukemia; 3) documented relapse after the first HSCT; 4) receipt of a second HSCT during the study period; and 5) availability of complete clinical records. Patients were excluded if they met any of the following criteria: 1) receipt of a second transplantation from a non-haploidentical donor, such as a matched sibling donor or an unrelated donor; 2) presence of an uncontrolled active infection or organ failure that precluded transplantation; 3) prior history of solid organ transplantation; or 4) incomplete follow-up data.

Eligibility for a second haploidentical transplantation was determined through an individualized multidisciplinary assessment. Relapse after the first transplantation was considered a prerequisite but was not by itself sufficient for second transplantation. The assessment considered disease status after salvage therapy, with at least partial remission or acceptable disease control required, adequate organ function, including left ventricular ejection fraction > 40%, and creatinine clearance > 50 mL/min, and absence of uncontrolled infection. Patients with early relapse, defined as relapse within 6 months after the first transplantation, were not automatically excluded; instead, they underwent individualized risk-benefit assessment before proceeding to second transplantation.

Comprehensive clinical data, including recipient parameters (sex and age at transplantation), donor characteristics (haploidentical/related/unrelated donor type, sex, and donor–recipient sex matching), disease profiles (baseline diagnosis, pre-second HSCT disease status), temporal dynamics (first transplantation-to-relapse interval, intertransplant duration), transplant protocol specifications (conditioning intensity [MAC/RIC], CD34 + cell dose [×10^6^/kg]), and immunological sequelae (acute/chronic GVHD [aGVHD/cGVHD] occurrence and severity grading), were extracted retrospectively from medical records.

The study protocol was conducted in accordance with institutional regulations and approved by the Ethics Review Committee of Shanghai Children’s Hospital (IRB Number: 2020R098-F02). The requirement for written informed consent was waived due to the retrospective nature of the research. All procedures were performed in compliance with the Declaration of Helsinki and applicable ethical standards for human research.

### 2.2 Conditioning regimens and donor selection

Three conditioning approaches were used. The TBI-based myeloablative conditioning regimen consisted of fractionated total body irradiation at 12 Gy, administered as 4 Gy/day for 3 days, fludarabine 30 mg/m^2^/day for 4 days, and cyclophosphamide 60 mg/kg/day for 2 days. The busulfan-based myeloablative regimen consisted of intravenous busulfan 3.2 mg/kg/day for 4 days, fludarabine 30 mg/m^2^/day for 4 days, and cyclophosphamide 50 mg/kg/day for 2 days. The reduced-intensity conditioning regimen consisted of busulfan 3.2 mg/kg/day for 2 days, fludarabine 30 mg/m^2^/day for 4 days, and thiotepa 10 mg/kg/day for 1 day. Regimen selection was based on institutional practice and patient characteristics. TBI-based conditioning was generally preferred for children aged > 4 years without prior radiotherapy and for patients with central nervous system involvement, whereas busulfan-based conditioning was used for younger children or those with prior TBI exposure.

For donor selection, a haploidentical donor different from the first transplant donor was preferred whenever feasible, which was achieved in 90% of cases. When more than one suitable donor was available, paternal donors were prioritized over maternal donors because of their potential immune tolerance advantage. If no suitable parental donor was available, siblings or other extended family members were considered.

### 2.3 GVHD prophylaxis

GVHD prophylaxis consisted of cyclosporine A, mycophenolate mofetil, and methotrexate. Cyclosporine A was initiated intravenously at 2.5 mg/kg/day from day -1, switched to oral administration at 12 mg/kg/day when tolerated (dose adjustments were individualized based on therapeutic drug monitoring and concomitant medications), and tapered from day +90 if no GVHD occurred. Mycophenolate mofetil was administered at 30 mg/kg/day, with a maximum dose of 2 g/day, from day 0 to day +30. Methotrexate was administered at 15 mg/m2 on day +0, followed by 10 mg/m2 on days +3, +6, and +11.

### 2.4 Response and clinical outcomes

We defined complete remission (CR) as the recovery of normal bone marrow cellularity with < 5% blasts, confirmed alongside the absence of disease-specific clinical manifestations upon physical examination. Relapse after the second allo-HSCT was defined as the presence of more than 5% blasts in bone marrow aspirates. Cases with minimal residual disease or molecular recurrence, in the absence of morphological abnormalities, were not considered relapsed.

Minimal residual disease was assessed by multiparameter flow cytometry using 8- to 10-color panels targeting leukemia-associated aberrant immunophenotypes. The sensitivity threshold of multiparameter flow cytometry was 0.01% (10^-4^), and minimal residual disease positivity was defined as abnormal cells accounting for ≥0.01% of total nucleated cells. For patients with known genetic abnormalities, such as fusion transcripts or mutations, quantitative PCR was also performed, with a sensitivity of 0.001% (10^-5^). Molecular minimal residual disease was evaluated before salvage therapy, within 14 days before conditioning for the second HSCT, on post-transplant days +30, +45, +60, and +90, every 3 months thereafter.

Survival outcomes including overall survival (OS), leukemia-free survival (LFS), and non-relapse mortality (NRM) were calculated. The primary endpoints were OS and LFS after the second allo-HSCT. OS was calculated from the date of the second allo-HSCT to the date of death from any cause or last follow-up. LFS was defined as the time from the second allo-HSCT to either hematologic relapse or death, whichever occurred first. NRM referred to deaths unrelated to disease relapse/progression. Molecular relapse was defined as the reappearance of leukemia-specific genetic markers, such as fusion transcripts including RUNX1-RUNX1T1, KMT2A, rearrangements, or NPM1 mutation, detected by quantitative PCR in two consecutive samples at least 2 weeks apart, in the absence of morphological relapse, defined as < 5% blasts in bone marrow. All time-to-event outcomes were measured from the date of the second allo-HSCT. Conditioning regimens were stratified as either myeloablative conditioning (MAC), defined by ≥ 8 Gy of total body irradiation (TBI) or > 8 mg/kg of busulfan based on actual body weight, or reduced-intensity conditioning (RIC), which included all other regimens. aGVHD and cGVHD were diagnosed and graded according to the NIH consensus criteria (8, 9). The GVHD prophylaxis regimen comprised cyclosporine A, mycophenolate mofetil, and short-course methotrexate administered according to standardized protocols.

### 2.5 Statistical analyses

We summarized continuous variables as with means ± standard deviations or medians with interquartile ranges (IQRs), depending on distribution, and compared them using the student’s t-test or Mann–Whitney U test, respectively. Categorical data were presented as counts and percentages, and analyzed using the chi-square test or Fisher’s exact test, as appropriate.

The survival curves for OS, LFS, NRM, and relapse were estimated using Kaplan–Meier method with log-rank test for intergroup comparisons. The median survival time and their 95% confidential intervals (95% CI) were calculated. The univariate analysis for detecting prognostic factors on 2-year OS across subgroups of recipient age, recipient sex, donor sex, donor–recipient sex compatibility, disease type, time from first transplantation to relapse, interval between transplants, morphological remission status before the 2^nd^ transplant, minimal residual disease before the 2^nd^ transplant, molecular status before the 2^nd^ transplant, conditioning regimen type, CD34 + cells dose, and occurrence of aGVHD or cGVHD were applied. We used multivariate Cox proportional hazards model to explore the potential predictors (univariate model P < 0.05 and the stepwise selected P < 0.05) for OS and calculated the hazard ratio for the predictors and their 95% CIs.

Statistical analyses were conducted systematically using SPSS v26.0 (IBM Corp.) and GraphPad Prism 9.0 (GraphPad Software). All P-values were two-tailed, and values < 0.05 were considered statistically significant.

## 3 Results

Totally 40 pediatric patients with a median age of 8.5 years (range: 2–18 years) were enrolled and underwent a second allo-HSCT (boys: 70.0%, n = 28 vs. girls: 30.0%, n = 12) (**Table 1**). Among them, acute myeloid leukemia (AML) as the predominant diagnosis (62.5%, n = 25), followed by acute lymphoblastic leukemia (ALL; 37.5%, n = 15) with subtype distribution (B-cell lineage: 22.5%, n = 9; T-cell lineage: 15.0%, n = 6).

**Table 1:** Summary of Patient Baseline Features.

Characteristics	Total (n=40)
Age (years), median (range)	8.5 (2-18)
Sex, n (%)	
Male	28 (70.0)
Female	12 (30.0)
Donor’s sex, n (%)	
Male	13 (32.5)
Female	27 (67.5)
Sex match status	
Mismatch	27 (57.5)
Match	13 (42.5)
Disease, n (%)	
AML	25 (62.5)
B-ALL	9 (22.5)
T-ALL	6 (15.0)
Duration of remission after the first HSCT (months), n (%)	
< 3	4 (10.0)
3 – 5.9	10 (25.0)
6 – 11.9	6 (15.0)
≥ 12	20 (50.0)
Time from relapse to the second HSCT (months), n (%)	
< 3	7 (17.5)
3 – 5.9	13 (32.5)
6 – 11.9	9 (22.5)
≥ 12	11 (27.5)
Time from the first HSCT to the second HSCT (months), n (%)	
< 3	0
3 – 5.9	2 (5.0)
6 – 11.9	10 (25.0)
≥ 12	28 (70.0)
Disease status at the second HSCT, n (%)	
CR (morphologically)	34 (85.0)
Non-CR	6 (15.0)
Donor type of the first HSCT, n (%)	
MSD	3 (7.5)
MUD	3 (7.5)
UCB	1 (2.5)
Haploidentical	33 (82.5)
Donor type of the second HSCT, n (%)	
Paternal donors	10 (25.0)
Maternal donors	24 (60.0)
Sibling donors	4 (10.0)
Extended familial donors	2 (5.0)
HLA mismatch status for the second transplant, n (%)	
5-loci mismatches	35 (87.5)
4-loci mismatches	3 (7.5)
3-loci mismatches	1 (2.5)
2-loci mismatches	1 (2.5)
Conditioning intensity for the first HSCT, n (%)	
MAC regimen	40 (100)
RIC regimen	0
Conditioning intensity for the second HSCT, n (%)	
MAC regimen	38 (95.0)
RIC regimen	2 (5.0)
Conditioning type for the second HSCT, n (%)	
TBI-based	21 (52.5)
TBI/Flu/Cy	14 (35.0
TBI/Cy	7 (17.5)
No TBI	19 (47.5)
Bu/Flu/Cy	11 (27.5)
DAC/Bu/Cy	2 (5.0)
Bu/Cy	2 (5.0)
Bu/Flu/melphalan	2 (5.0)
RIC-Bu/Flu/thiotepa	2 (5.0)
Source of stem cell, n (%)	
PBSC+BM	21 (52.5)
PBSC	19 (47.5)
The median total nucleated cell dose, 10^8^/kg, median (IQR)	10.77 (4.50–18.74)
The median CD34 + cell dose, 10^6^/kg, median (IQR)	4.53 (1.6–16.45)
Neutrophil engraftment, day, median (IQR)	13 (10–41)
Platelet engraftment, day, median (IQR)	14 (7–44)

Among these children, the first transplantation involved haploidentical donors in 33 patients (82.5%), matched sibling donors in 3 patients (7.5%), and unrelated donors in 4 patients (10%). The second donor sources were distributed as follows: paternal donors in 10 cases (25%), maternal donors in 24 cases (60%), sibling donors in 4 cases (10%), and extended familial donors in 2 cases (5%), including one maternal uncle and one paternal aunt. HLA disparity analysis in the graft-versus-host direction demonstrated significant antigenic mismatches: 5-loci mismatches were the highest (87.5%, n = 35), followed by 4-loci mismatches (7.5%, n = 3), 3-loci mismatches (2.5%, n = 1), and 2-loci mismatches (2.5%, n = 1) (**Table 1**). Nearly half of children (n=20) had a duration of remission after the 1^st^ HSCT longer than 12 months, 15% (n=6) had 6–12 months, 25% had 3–6 months (n=10), and 10% (n=4) had < 3 months.

The intervals from relapse to the second allo-HSCT was less than 3 months in 7 patients, 3–6 months in 13 patients, 6–12 months in 9 patients, and ≥12 months in 11 patients (**Table 1**). The median age at the time of the 2^nd^ transplantation was 8.5 years (IQR: 2–18 years), and the median time from relapse to the 2^nd^ HSCT was 5.5 months (IQR: 1.6–35.8 months).

Post-transplant therapeutic interventions were limited. One patient with AML received venetoclax maintenance therapy, while 4 AML patients without CR before HSCT received prophylactic decitabine. The majority of patients (87.5%, n = 35) did not receive any novel targeted therapies after transplantation.

The median interval between the 1st and 2^nd^ HSCT was 20.7 months (IQR: 4.0–69.5 months). Donor selection and conditioning regimens tailored to donor availability and individual clinical status. Institutional protocols emphasizing the utilization of alternative donors resulted in 90% (36/40) of patients receiving grafts from different donors for their 2^nd^ transplantation.

Myeloablative conditioning was administered to 38 patients (95.0%), while reduced-intensity conditioning was used only in 2 patients (5%) with pre-existing organ dysfunction. Among those receiving myeloablative conditioning, 52.5% (n=21) underwent total body irradiation (TBI)-based regimens, including TBI/fludarabine [Flu]/cyclophosphamide [Cy] in 14 cases and TBI/Cy in 7 cases. Busulfan (Bu)-based regimens were used in 42.5% (17/40), including Bu/Flu/Cy (n = 11), DAC/Bu/Cy (n = 2), Bu/Cy (n = 2), and Bu/Flu/melphalan (n = 2). The two patients receiving RIC administered Bu/Flu/thiotepa. All pediatric patients received a standardized dose of rabbit anti-human thymocyte globulin (ATG; 10.0 mg/kg, Sanofi, France) as part of the conditioning protocol.

Stem cell sources included combined bone marrow and peripheral blood in 21 patients (52.5%) and peripheral blood alone in 19 patients (47.5%). The median total nucleated cell dose was 10.77 × 10^8^/kg (IQR: 4.50–18.74), and the median CD34 + cell dose was 4.53 × 10^6^/kg (IQR: 1.6–16.45). All patients achieved neutrophil engraftment at a median of 13 days (IQR: 10–41), and platelet recovery occurred in 35 patients (87.5%), with a median time of 14 days (IQR: 7–44). Complete donor chimerism was confirmed in all patients at 30 days after transplant. Acute GVHD developed in 40.0% (n=16) of patients, including 15.0% (n=6) with grade I–II and 25.0% (n=10) with grade III–IV. Chronic GVHD occurred in 50.0% (n=20) of patients, with most cases being limited (40.0%, n=16) and 10.0% (n=4) classified as extensive (**Table 1**).

**Figure 1:**
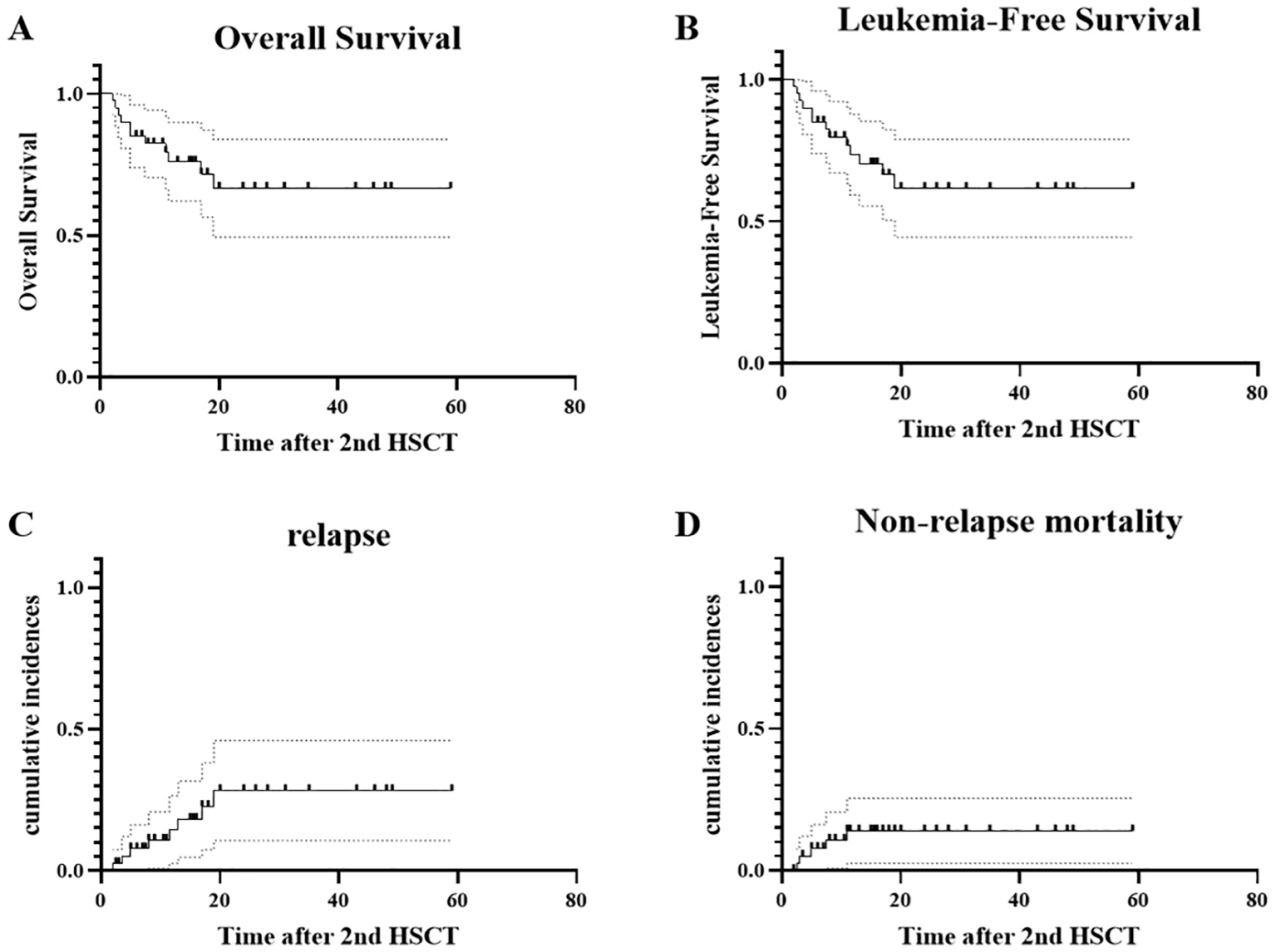
Outcomes after the second hematopoietic stem cell transplantation (HSCT), which was a haploidentical-HSCT (haplo-HSCT) **(A)** Overall survival (OS). **(B)** Leukemia-free survival (LFS). **(C)** Relapse. **(D)** Non-relapse mortality (NRM). The Kaplan–Meier method was used to analyze OS and LFS.

A total of 11 patients died during a median follow-up time of 16.0 months (IQR: 2.5–61). The leading cause of death was disease relapse/progression, accounting for 6 deaths due to leukemia relapse. The median time to relapse was 4.5 months (IQR: 1.5–12), and the median post-relapse survival was 8.5 months (IQR: 2–19). The remaining 5 deaths were attributed to NRM, including septic shock (n=2), post-transplant lymphoproliferative disorder (n=1), diffuse alveolar hemorrhage (n=1), and cerebral hemorrhage (n=1). The median survival time in NRM cases was 5 months (IQR: 2.5–11). Among the 29 surviving patients, 2 with AML exhibited molecular relapse and are currently receiving oral venetoclax maintenance therapy combined with DAC prophylaxis. Kaplan–Meier revealed a 1-year OS rate of 77.8% (95% CI: 70.8-85.8) and a 2-year OS rate of 65.5% (95%CI: 56.7-74.3). The 1-year and 2-year LFS rates were 72.4% (95%CI:64.9-79.9) and 61.0% (95%CI:52.2-69.8), respectively. The cumulative incidence of relapse was 14.2% (95% CI: 8.3-20.1) at 1 year and 26.6% (95%CI:18.1-35.1) at 2 years, whereas the cumulative incidence of NRM remained stable at 15.1% (95% CI: 8.9-21.3) at both 1 and 2 years (**Figure 1**). Regarding the prognosis across different subgroups, univariate analysis grouped patients by recipient age, recipient gender, donor gender, donor-recipient gender match, disease type, time from first transplant to relapse, interval between transplants, morphologic remission status before the second transplant, flow cytometry minimal residual disease (MRD) status before the second transplant, molecular status before the second transplant, conditioning regimen type, number of CD34+ cells infused, aGVHD, cGVHD, and post-transplant interventions. Ultimately, it was found that disease type, morphologic remission status before the second transplant, and occurrence of aGVHD showed statistically significant differences (P < 0.05) in OS (**Table 2**). Multivariate analysis identified that disease type (B-ALL) was an independent risk factor associated with 2-year OS (**Table 3**), the HRs were 0.081 (95% CI: 0.007, 0.877, P = 0.039). While aGVHD and AML were marginally associated with risk of 2-year OS with the HRs of 0.120 (95% CI: 0.013, 1.08, P = 0.059) and 0.208 (0.042, 1.038, P = 0.056).

**Table 2:** Single-factor analysis of survival determinants in the study cohort Univariate analysis of the risk factors for OS.

Risk factors	n (%)	2-year OS (95% CI)	P value
Recipient’s age			
≥10 years	17(42.5)	66.6(52.2-81.0)	0.498
<10 years	23(57.5)	65.7(54.8-76.6)	
Recipient’s sex			
Male	28(70.0)	65.7(55.4-76.0)	0.969
Female	12(30.0)	69.4(53.9-84.9)	
Donor’s sex, n (%)			
Male	13(42.5)	47.6(29.7-65.5)	0.313
Female	27(57.5)	74.3(65.0-83.6)	
Sex match			
Mismatch	27(57.5)	60.2(43.4-77.0)	0.706
Match	13(42.5)	50.55(32.8-68.3)	
Disease entity			
AML	25(62.5)	66.5(53.6-79.4)	0.034
B-ALL	9(22.5)	87.5(75.8-99.2)	
T-ALL	6(15.0)	33.3(14.1-52.5)	
Time from the first HSCT to relapse			
≥6 months	26(65.0)	60.6(49.0-72.2)	0.522
<6 months	14(35.0)	75.0(62.1-87.9)	
Time from the first to the second HSCT, months			
≥12	28(70.0)	58.8(47.4-70.2)	0.457
≥6 to < 12	10(25.0)	77.1(62.7-91.5)	
≥3 to < 6	0	N/A	
Bone marrow morphology status before the second HSCT			
CR	37(92.5)	69.1(68.2-70.0)	0.031
Non-CR	3(7.5)	33.3(6.1-60.5)	
Cytogenetic status before the second HSCT			
FCM-negative	24(60.0)	66.1(55.4-76.8)	0.717
FCM-positive	16(40.0)	65.6(50.8-80.4)	
Molecular Biology Status before the second HSCT			
Gene-negative	23(57.5)	65.0(52.9-77.1)	0.635
Gene-positive	17(42.5)	65.5(52.7-78.3)	
Conditioning			
MAC regimen	38(95.0)	67.2(58.3-76.1)	0.345
RIC regimen	2(5.0)	50.0(14.6-85.4)	
Conditioning including TBI			
Yes	21(52.5)	58.0(45.4-70.6)	0.386
No	19(47.5)	73.9(62.1-85.7)	
CD34+stem cell dose			
>10.0× 10^6^/kg	4(10.0)	75.0(53.3-96.7)	0.578
<10.0× 10^6^/kg	36(90.0)	65.8(56.6-75.0)	
CD34+stem cell dose			
>5.0× 10^6^/kg	18(45.0)	69.1(55.5-82.7)	0.526
<5.0× 10^6^/kg	22(55.0)	62.6(51.2-74.0)	
Acute GVHD			
No	24(60.0)	53.5(41.9-65.1)	0.046
Yes	16(40.0)	88.9(78.4-99.4)	
Acute GVHD			
Grade 0–II	30(75.0)	74.4(65.1-83.7)	0.072
Grade III–IV	10(25.0)	40.9(23.2-58.6)	
Chronic GVHD			
No	20(50.0)	71.1(59.5-82.7)	0.705
Localized	16(40.0)	69.2(56.0-82.4)	
Extensive	4(10.0)	50.0(25.0-75.0)	
Post-transplant therapeutic interventions			
Yes	5(12.5)	80.0(62.1-97.9)	0.615
No	35(87.5)	67.8(58.4-77.2)	

OS overall survival; CR complete remission.

**Table 3:** Multivariate analysis of the risk factors for OS.

Disease type	HR	95% CI		P
		Lower limit	Upper limit	
AML	0.208	0.042	1.038	0.056
ALL	0.081	0.007	0.877	0.039
Bone marrow morphology status before the second HSCT	4.093	0.693	24.16	0.120
Acute GVHD (Yes or No)	0.120	0.013	1.080	0.059

*P < 0.05 significant statistical difference.

## 4 Discussion

This retrospective study included 40 pediatric patients with AL who relapsed after a 1^st^ allo-HSCT. Our findings suggest that haplo-HSCT may be an effective salvage strategy in this setting. The 1- and 2-year OS rates were 77.8% (95% CI: 70.8-85.8) and 65.5% (95%CI: 56.7-74.3), while the corresponding LFS rates were 72.4% (95%CI:64.9-79.9) and 61.0% (95%CI:52.2-69.8), respectively. Notably, 85.8% of patients remained free from relapse within the 1^st^ year after the 2^nd^ transplant, suggesting that the early relapse-free survival may be associated with sustained clinical benefits.

Leukemia relapse remains a major critical clinical challenge that significantly compromises long-term survival in pediatric patients, particularly in those who relapse after a 1^st^ allo-HSCT. Thus, the development of effective salvage strategies has become a critical focus in hematologic oncology. In our study, haplo-HSCT as a second transplant demonstrated encouraging outcome, with survival rates appearing favorable compared with previously reported 5-year OS rates of 24%–42% and disease-free survival rates of 24%–44% (10–14), although cross-study comparisons should be interpreted cautiously. Recent studies have further supported second allo-HSCT as a feasible but high-risk salvage strategy, emphasizing the importance of conditioning intensity and transplant-related toxicity (15–20). These findings are broadly consistent with our results and highlight the importance of careful patient selection and optimized peri-transplant management.

Current evidence indicates that achieving CR before the 2^nd^ allo-HSCT is a strong prognostic factor. Patients in CR at the time of transplant generally show significantly improved outcomes (11, 21, 22). For instance, Japanese cohorts have reported a 2-year OS rate of 86.7% in pediatric patients who achieved CR prior to the 2^nd^ transplant, compared to only 31.7% in those who did not (2, 23). In our study, univariate analysis similarly confirmed that patients in CR before 2^nd^ transplant had significantly higher 2-year OS than those with active disease (69.1% vs. 33.3%, p = 0.031). For pediatric patients with molecular residual disease (MRD) prior to 2^nd^ transplant, no statistically significant survival difference was observed. One possible explanation may be the implementation of systematic prophylactic strategies (e.g., DAC). Such targeted interventions may have partly mitigated the adverse prognostic impact of pre-transplant non-remission status, thereby narrowed the survival disparity and improved OS outcomes in this high-risk subgroup.

Multivariate analysis identified disease subtype and occurrence of aGVHD as independent prognostic factors for OS. Among disease subtypes, patients with B-ALL demonstrated the most favorable outcomes, followed by those with AML and T-ALL. This survival gradient should be interpreted cautiously. One possible explanation is that patients with B-ALL may benefit from advances in pre-second-transplant disease control, including CAR-T therapy, blinatumomab, and other immunotherapeutic approaches (24, 25). However, the present study did not directly evaluate the independent effects of these therapies, and therefore the favorable survival observed in the B-ALL subgroup cannot be specifically attributed to CAR-T therapy or immunotherapy. Currently, standardized therapeutic strategies for relapsed leukemia before 2^nd^ allo-HSCT remain inadequately supported by high-quality evidence. Therefore, future research should clarify how targeted agents and immunotherapies may improve pre-transplant remission depth and post-transplant outcomes in this high-risk population.

The role of GVHD in shaping post-transplant outcomes remains complex. Although prior studies have suggested that acute GVHD may be associated with improved disease-free survival through graft-versus-leukemia effects (21), others have reported no clear survival benefit after a second transplant (26). Our study suggested that acute GVHD may be associated with a protective graft-versus-leukemia effect, consistent with adult data linking post-transplant alloreactivity to improved survival (21). However, this association should be interpreted cautiously according to GVHD severity, as patients with grade III-IV acute GVHD showed numerically inferior 2-year OS compared with those with grade 0-II acute GVHD. Thus, any potential antileukemic benefit of acute GVHD should not be extrapolated to severe acute GVHD, which may increase treatment-related morbidity and mortality. Furthermore, prior studies have shown that adult patients with acute or chronic leukemia who developed chronic GVHD after a 2^nd^ transplant often experience favorable long-term outcomes, possibly due to a sustained graft-versus-leukemia effects balanced against GVHD severity (7, 27, 28). Despite a high incidence of chronic GVHD (50%) in our cohort, survival was not significantly affected, possibly owing to the limited median follow-up of 16.0 months. Precise modulation of GVHD may therefore be essential for balancing sustained remission, treatment-related toxicity, and quality of life after transplant.

Our study did not identify a significant association between the interval from the 1^st^ allo-HSCT to leukemia relapse and OS, suggesting that early relapse (< 6 months) is not an absolute contraindication to proceeding with a second transplantation. Previous reports have indicated that patients undergoing a second transplantation within 6 months of their initial allo-HSCT experience significantly poorer outcomes than those with longer relapse-free intervals (29, 30). In our cohort, only 5% of children received a 2^nd^ transplant within 6 months of the first. Although no statistically significant difference in survival was observed between patients with shorter versus longer inter-transplant intervals, the early-transplant subgroup exhibited a trend toward lower 2-year OS rates. These findings imply that while early relapse does not preclude the possibility of a 2^nd^ transplant, a longer interval between transplants may contribute to more favorable outcomes. This benefit may be mediated by improved graft-versus-leukemia effects or by allowing time for recovery of the recipient’s hematopoietic and immunologic microenvironment, thereby increasing tolerance to conditioning regimens and enhancing graft function.

We found no significant association between conditioning intensity (MAC vs. RIC) and overall survival. In our cohort, 95% of patients received MAC, whereas 5% underwent RIC; all RIC patients died from disease progression, with median survival of 11 months. Although RIC is theoretically expected to lower non-relapse mortality and improve survival, neither our data nor prior pediatric studies confirmed this benefit (22, 23, 31). This may reflect children’s greater physiological tolerance of MAC, supported by the low NRM rate in our MAC group (13.2%), which was lower than that reported in adult studies (14, 26, 32–34). Pediatric patients may benefit from superior organ reserve and enhanced regenerative capacity, mitigating MAC-related toxicity. Additionally, 3-year OS did not differ between TBI-based and Bu-based MAC, indicating that Bu-based regimens may be viable alternative for centers without TBI resources.

This study has several limitations that warrant consideration. First, the retrospective design may introduce selection bias and limits the ability to establish causality. Second, the sample size was relatively small, especially in certain subgroups such as patients receiving RIC or early second transplants, which may reduce the statistical power to detect meaningful differences in survival outcomes. Third, the follow-up duration was relatively short, with a median follow-up of 16 months, potentially underestimating long-term complications such as late relapse, chronic GVHD progression, or delayed treatment-related mortality. Additionally, heterogeneity in disease subtypes, prior therapies, and post-transplant interventions, such as maintenance strategies, may have influenced outcomes and limited the generalizability of our findings. Finally, molecular and immunological data, such as measurable residual disease kinetics, donor chimerism dynamics, or immune reconstitution profiles, were not comprehensively assessed, which may have provided further insights into prognosis and treatment response. Further multicenter prospective studies with larger cohorts, longer follow-up, and standardized therapeutic protocols are needed to validate and expand upon these findings.

In conclusion, haplo-HSCT is a feasible and effective salvage strategy for pediatric acute leukemia relapsing after allo-HSCT, with favorable survival in selected patients who remain event-free early after transplantation. While pretransplant remission remains important, tailored post-transplant strategies may offset its impact in some high-risk patients. The tolerability of myeloablative conditioning and the comparable efficacy of busulfan- and TBI-based regimens support broader applicability. Future work should prioritize optimizing remission with targeted agents and immunotherapies, prospectively evaluating their independent contribution to transplant outcomes, and refining GVHD prophylaxis and immune modulation to improve long-term survival.

## Data Availability

The raw data supporting the conclusions of this article will be made available by the authors, without undue reservation.
